# Robotics in Colorectal Surgery

**DOI:** 10.12688/f1000research.9389.1

**Published:** 2016-09-26

**Authors:** Allison Weaver, Scott Steele

**Affiliations:** 1 Case Western Reserve University School of Medicine, Cleveland, OH, USA; 2 Department of Surgery, University Hospitals Case Medical Center, Cleveland, OH, USA

**Keywords:** robotic surgery, colorectal cancer, haptic feedback

## Abstract

Over the past few decades, robotic surgery has developed from a futuristic dream to a real, widely used technology. Today, robotic platforms are used for a range of procedures and have added a new facet to the development and implementation of minimally invasive surgeries. The potential advantages are enormous, but the current progress is impeded by high costs and limited technology. However, recent advances in haptic feedback systems and single-port surgical techniques demonstrate a clear role for robotics and are likely to improve surgical outcomes. Although robotic surgeries have become the gold standard for a number of procedures, the research in colorectal surgery is not definitive and more work needs to be done to prove its safety and efficacy to both surgeons and patients.

## Introduction

Over the past few decades, the surgical field has undergone some of the most revolutionary shifts in modern medicine. The invention and widespread acceptance of laparoscopic approaches across all surgical disciplines were among the most significant advances and they are now the gold standard for a range of procedures. The incorporation of robotics into a minimally invasive surgery platform is the newest advancement and has the potential to change the medical field even more drastically with the minimization—and possible elimination—of human error. However, further advancement in this field has been limited by a plethora of challenges that must be addressed, including high costs, difficult implementation, and still somewhat limited technologies.

Despite significant evidence supporting its safety and efficacy, colorectal surgery has been relatively slow to adapt to minimally invasive surgical techniques. The COST (Clinical Outcomes of Surgical Therapy) and COLOR (Colon Carcinoma Laparoscopic or Open Resection) trials successfully demonstrated the non-inferiority of laparoscopic approaches for colectomy, and later trials also demonstrated superior cosmesis, lower conversion to open rates, faster return of bowel function, and shorter hospital stays ^1– 5^. Although these benefits would seem to denote superiority of a minimally invasive approach, the adoption of these techniques has been slow, and since the publication of the COST trial in 2004, only about 55% of colonic tumor resections are done laparoscopically ^6^. This resistance can be attributed to a variety of obstacles, including inadequately trained surgeons, poor standardization of procedures, and an inability for the technology to overcome anatomic restrictions, especially in rectal procedures where narrow pelvises and obese abdomens are limiting factors ^7^. Although robotics offers probable advantages in a number of laparoscopic procedures, including benign diseases like diverticulitis and inflammatory bowel disease, rectal procedures in which enhanced maneuverability in the confined space of the pelvis is needed are the most likely beneficiaries ^8^.

Thus, traditional laparoscopic procedures are limited and a variety of approaches have attempted to compensate for these restrictions. This article will address the potential advantages, as well as shortcomings, of the development of robotic procedures and their potential for improvement of both open and minimally invasive colorectal surgeries.

## Robotic technology

Robotic surgery has transformed and advanced as new technologies have made it possible to compensate for different aspects of innate human error and expand our capabilities. Currently, the da Vinci Robot by Intuitive Surgical (Sunnyvale, CA, USA) is the most widely used model for robot-assisted laparoscopic procedures since being approved by the US Food and Drug Administration in 2000. The surgical system consists of a control module with a high-definition, three-dimensional (3D) camera where the surgeon sits and controls effector robotic arms in a “master-slave” setup ^9^. Each system has an endoscope and three to four effector arms that attach EndoWrist devices, or interchangeable instruments that can be manipulated with greater maneuverability than the human wrist. While new systems are currently in development and will likely result in both economic and technology competition, the da Vinci remains the leader in robotic surgical technology.

Since its approval for general abdominal procedures in 2000, the da Vinci robotic system has been approved for an increasing number of procedures ^10^. Although it has largely overtaken other minimally invasive techniques for prostate resection ^11^, it has yet to gain the same traction in general surgery. However, laparoscopic surgeries are still uncommon for rectal procedures and conversion rates to open surgeries are still high ^12^. The Society of American Gastrointestinal and Endoscopic Surgeons released its official recommendation for the da Vinci, endorsing it as safe and effective for rectal procedures but citing its significant cost and lack of proven superiority as reasons for limiting its use ^13^. Going forward, it will be important to address the limitations of traditional minimally invasive surgery in rectal resections and procedures and how robotics can be applied to address these issues.

## Advantages of robotics

Robotic surgery presents a spectrum of advantages over traditional laparoscopic techniques. Robotics allows the surgeon to see 3D images, obtain better angles with the increased degrees of freedom provided by the EndoWrist, and control three different instruments simultaneously. These advantages make it easier to execute complex laparoscopic procedures like identifying important neurovascular structures and intracorporeal suturing in a deep and narrow pelvis ^14^. Additionally, the newer da Vinci systems are able to filter tremor, effectively eliminating a major source of human error ^15^. These technological improvements present new opportunities for surgeons to improve the way surgery is performed.

As with any new technology, there is a significant learning curve for adapting to the new procedures and techniques. However, several studies have demonstrated that this learning curve is no worse than that of traditional laparoscopic surgery and that in surgeons already trained in laparoscopic surgery this mastery can be achieved in about 30 cases ^16, 17^. One barrier to attaining this proficiency requires that the surgeon have a large enough patient population to perform this number of procedures, and this may not always be possible. In addition, the time frame over which consecutive cases can be performed has an effect. Therefore, performing 50 cases may be feasible but over several years this decreases the impact of acquiring the necessary skills to attempt to master the technique. Also, the loss of tactile sensation can present large challenges in developing proficiency in minimally invasive techniques for surgeons who were trained in traditional methods. Intuitive Surgical has been attempting to compensate for these difficulties by developing surgical simulators and a dual console system that offer new methods to train surgeons ^18^. Training and credentialing programs have been set up by some institutions to standardize teaching robotics and may be necessary to increase the number of residents and surgeons who feel comfortable performing robotic techniques ^19, 20^. In any case, the current evidence suggests that there is no overwhelming barrier to learning robotic techniques above laparoscopy and that, with the development of new technologies, it might be even easier than traditional laparoscopic techniques ^21^.

These technological attributes speak to the potential of robotic systems, but the real test of efficacy is their ability to improve clinical outcomes. Results from a number of recent studies are summarized in Table 1. Early studies were very promising, finding lower conversion to open rates and faster return of bowel function ^22^. Studies of total mesorectal excision, the current mainstay of treatment for low rectal cancers, found that robotic surgeries had fewer genitourinary complications and lower rates of positive circumferential margins, a measurement of pathological success, and that other outcomes were equal to those of traditional laparoscopic surgery ^23^. However, the success of these early trials is likely to be tempered by the new data from the ROLARR (Robotic versus Laparoscopic Resection for Rectal Cancer) trial, the first multicenter trial comparing robotic versus laparoscopic rectal cancer excisions ^24^. This study represented the work of 40 surgeons from 10 countries and 29 hospitals randomly assigning 471 patients with rectal cancer to laparoscopic versus a robotic approach. Though not yet published, early data presented at an annual meeting of the American Society of Colon and Rectal Surgeons fail to prove superiority of robotics with regard to a number of variables, including circumferential margin positivity, 30-day complication rates, and mortality. There was some evidence of lower conversion to open rates, usually an indicator of decreased complication rates, and possible benefits in males and the obese. Some of these results are supported by other studies, suggesting that male gender and obesity may be strong indicators for robotic surgery moving forward ^25, 26^. The study design was constructed as a superiority trial (for conversion), thus causing some controversy regarding the take-home message depending on the point of view of the beholder. For proponents, these data represented equivalent outcomes between both approaches. How opponents—and, for that matter, payers and patients—view these results remains to be determined.

**Table 1.** Recent studies on robotic versus laparoscopic or open rectal cancer excision outcomes.

Reference	Surgery	Number of patients	Conversion rate	Positive circumferential resection margin	Operative time, minutes	Complication rate
Bianchi *et al*. ^58^ (2011)	Robot	25	0%	0%	240	16%
	Lap	25	4%	4%	237	24%
D’Annibale *et al*. ^59^ (2013)	Robot	50	0%	0%	270	10%
	Lap	50	12%	12%	280	22%
Ghezzi *et al*. ^60^ (2014)	Robot	65	1.5%	0%	299	41.5%
	Open	109	-	1.8%	207.5	41.3%
Yamaguchi *et al*. ^61^ (2015)	Robot	203	0%	0%	232.9 ± 72.0	8.9%
	Lap	239	3.3%	1%	227.6 ± 62.6	34%
Kim *et al*. ^62^ (2015)	Robot	33	6.1%	16.1%	441	45.6%
	Lap	66	0%	6.7%	227	39.4%
Cho *et al*. ^63^ (2015)	Robot	278	0.4%	5%	361.6 ± 91.9	25.9%
	Lap	278	0.7%	4.7%	272.4 ± 83.8	23.7%
Allemann *et al*. ^64^ (2015)	Robot	20	5%	10%	291	40%
	Lap	40	20%	25%	313	35%

Overall, there is evidence to promote the continued use of robotics in colorectal surgery. The ease with which it is adapted and expanded depends largely on the continued improvement of the technology and the ability to overcome a number of barriers to the use and acquisition of the equipment.

## Disadvantages of robotics

Though promising, many of the benefits of robotic surgeries still exist in its potential. If the barriers to the adoption and technical advancements are not overcome, the entire field may never reach its full capabilities. Higher costs, increased surgical time, loss of tactile sensation, and the inability for researchers to prove its superiority in a number of trials (especially across all institutions and not just in the hands of experts) must all be addressed.

A major handicap for surgeons interested in learning to use a surgical robot is the loss of tactile sensation that is of significant importance in a number of procedures. The job of a surgeon who is not able to feel the different tissues and tension becomes considerably more difficult, and the risks of perforation and injury increase ^27^. In addition, while “hand-sewn” techniques are more readily performed with robotics over laparoscopy, the tissue tension on the sutures needs to be gathered from visual cues. However, these barriers also exist in traditional laparoscopic approaches and it is possible that new robotic technology may be able to overcome them.

One of the most noticeable obstacles is the overwhelming cost associated with purchasing, maintaining, and operating the robotic system. Currently, Intuitive Surgical has very little competition in the robotic surgery field, allowing them to maintain high costs. Even after a hospital purchases the extremely expensive system, it is faced with higher per-surgery costs attributed to expensive instrumentation and disposables and longer operating times ^28^. These costs are ameliorated slightly by possible reductions in hospital stay and lower conversion to open surgery ^27^. However, studies have failed to prove that robotic surgery was cost-effective with regard to short-term outcomes in rectal cancer excision ^29^. As it stands, robotic procedures are significantly more expensive, and these costs must be either covered by the hospital or passed on to patients and insurance companies ^30, 31^. Ethically, it may be difficult for surgeons or hospitals to justify performing these more costly procedures when no definite advantage has been established.

Going hand-in-hand with increased costs, the longer operative times could have a negative impact on the adoption of robotic surgery. Studies have found that robotic surgery takes longer by varying degrees, and this increased operative time only serves to drive up costs ^32, 33^. This also means that surgeons can perform fewer procedures and that, given the limited access to the technology, fewer surgeries can be performed robotically. Most importantly, these longer operative times do not correlate with improved outcomes, making robotic surgery even less desirable ^34^.

Although the current barriers to robotic surgery are definitely surmountable, more needs to be done to justify its use and improve the technology. There is evidence that robotic colorectal procedures offer reduced blood loss, shorter length of stay, and shorter return of bowel function with similar complication rates when compared with open surgeries ^35^. However, for many procedures, no conclusive evidence has been able to prove any superiority of robotic surgery over laparoscopic approaches ^36, 37^. Whereas some researchers have been able to demonstrate benefits for rectal cancer excisions ^38^, most research has not been able to replicate these results ^37, 39^. But this does not mean that the future of robotic surgery is hopeless—the technology will continue to grow and develop and the research outcomes will likely reflect these advances.

## The future of robotic technology

Advances in robotic colorectal surgery are limited by the speed of technological discovery and the willingness of surgeons to adapt to these new devices and procedures. New robotic technologies could become the future of surgery with cheaper systems and new developments such as haptic and tactile sensing technology, miniature *in vivo* robots, and novel single-port laparoscopic approaches.

Moving forward, decreasing costs will be a major contributor to the widespread adoption of robotic surgery. Hospitals that can increase the number of robotic procedures performed can reduce per-case costs, and this can be accomplished by increasing the number of surgeons who perform robotics procedures and having a dedicated nursing team (which can also improve operating time) ^40^. However, these modifications will not reduce the costs of purchasing and maintaining the robotic system. Since purchasing Computer Motion in 2003, Intuitive Surgical has had a virtual monopoly on the robotic surgery industry ^41, 42^. Now, companies like TransEnterix (Morrisville, NC, USA), Titan Medical (Toronto, ON, Canada), and Virtual Incision (Pleasanton, CA, USA) are developing new systems and there is hope that this new competition will not only drive down prices but also lead to new technological innovation ^43^. In addition, similar to the early stages of laparoscopy for colorectal surgery, training well-versed surgeons (that is, senior surgeons already trained in open surgery and laparoscopy) the nuances of the robotic technique will have second- and third-tier effects that ultimately may lead to improved outcomes and increased utilization. Once more of these senior surgeons are performing more of their cases robotically and robotic use becomes a standard part of the curriculum of most trainees, the robotic approach may simply become another tool in the armamentarium of colorectal surgeons and not a divisive issue.

Several of the new robotic systems have focused on improving existing systems and incorporating new technology. The Telelap Alf-X by Sofar (Milan, Italy) was one of the first and most successful of this new generation and was recently purchased by TransEnterix. The setup is similar to that of the da Vinci with a control unit and patient sidecart with four maneuverable arms. Unlike the da Vinci, this system has an eye-tracking camera that follows the surgeon’s gaze and keeps the camera centered throughout the surgery ^44^. It also attempts to address the cost concerns of robotics by allowing the arms to attach reusable endoscopic instruments with magnets ^45^. But, most significantly, this is the first system to incorporate haptic feedback technology. As discussed, the inability for surgeons to feel tissues and the pressure exerted on their instruments is a major drawback in the da Vinci system. The Telelap Alf-X addresses this problem by providing direct force feedback that allows the surgeon to feel the instrument in their hands, sense the force applied, and palpate the texture of the tissues ^46, 47^. This is an enormous advancement for robotic surgery and could significantly improve the learning curve and the outcomes as surgeons are able to perform much more delicate movements. Early research with this system in gynecological surgeries has been very successful and its adoption could help drive down upfront and per-procedure costs ^46, 48^.

Similarly, some systems have been trying to take advantage of the improved maneuverability of robotics to optimize the technology for single-port surgeries. Single-port approaches have the potential to minimize complications and cosmetic concerns that are associated with traditional multi-port procedures ^49^. Da Vinci developed a Single-Site system that is compatible with their previous Si system that allows access through the umbilicus for several abdominal procedures ^50^. The system helps to prevent crowding and external collision of the instruments, which are curved and non-wristed to reach the surgical site from the single port. The new SPORT system by Titan Medical has not yet been released but is intended to present a cheaper alternative to the da Vinci system and is optimized for single-port access ^51^. This system—the Single Port Orifice Robotic Technology—has a 3D camera and snake-like instrument maneuverability. Early studies have shown longer operating times but low complication rates and improved cosmesis ^52, 53^. As the research develops, single-port surgery could become one of the biggest advantages of robotic surgeries.

Just as single-port surgeries are associated with fewer complications, natural orifice translumenal endoscopic surgeries (NOTES) are even less invasive. There has been some success in using NOTES for transanal excision of rectal masses, but no large-scale study has yet been done to compare it with non-robotic approaches ^54^. Unfortunately, current robotic technology is not optimized for these procedures and the instruments often are too large and have poor visualization. One approach has been to develop miniature *in vivo* robots that can be introduced to the body through natural orifices and perform unique tasks such as lighting, imaging, retraction, and biopsy ^55^. Currently, some of these devices are too large and still require an incision, indicating a need for further miniaturization ^56^. These devices have been used successfully in porcine models and could represent a novel approach to minimally invasive robotic colorectal surgery.

## Conclusions

The future of robotic surgeries is constantly evolving. The development of these new technologies and potentially cheaper systems opens the door to further research and new approaches. Everything from nanobots to artificially intelligent robots has been proposed and could be incorporated into future technology ^57^. New generations of surgeons will receive earlier and broader training in these techniques and be more comfortable with their application. However, the technology is still lacking and a number of obstacles must be addressed before robotics can truly match the costs and outcomes of earlier approaches.

## References

[1] Colon Cancer Laparoscopic or Open Resection Study Group, Buunen M Veldkamp R: Survival after laparoscopic surgery versus open surgery for colon cancer: long-term outcome of a randomised clinical trial. *Lancet Oncol.* 2009;10(1):44–52. 10.1016/S1470-2045(08)70310-3 19071061

[2] Veldkamp R Kuhry E Hop WC: Laparoscopic surgery versus open surgery for colon cancer: short-term outcomes of a randomised trial. *Lancet Oncol.* 2005;6(7):477–84. 10.1016/S1470-2045(05)70221-7 15992696

[3] Dunker MS Bemelman WA Slors JF: Functional outcome, quality of life, body image, and cosmesis in patients after laparoscopic-assisted and conventional restorative proctocolectomy: a comparative study. *Dis Colon Rectum.* 2001;44(12):1800–7. 10.1007/BF02234458 11742165

[4] Moghadamyeghaneh Z Hanna MH Carmichael JC: Comparison of open, laparoscopic, and robotic approaches for total abdominal colectomy. *Surg Endosc.* 2016;30(7):2792–8. 10.1007/s00464-015-4552-8 26487196

[5] Morneau M Boulanger J Charlebois P: Laparoscopic versus open surgery for the treatment of colorectal cancer: a literature review and recommendations from the Comite de l'évolution des pratiques en oncologie. *Can J Surg.* 2013;56(5):297–310. 10.1503/cjs.005512 24067514 PMC3788008

[6] Moghadamyeghaneh Z Carmichael JC Mills S: Variations in Laparoscopic Colectomy Utilization in the United States. *Dis Colon Rectum.* 2015;58(10):950–6. 10.1097/DCR.0000000000000448 26347967

[7] Champagne BJ Delaney CP: Laparoscopic approaches to rectal cancer. *Clin Colon Rectal Surg.* 2007;20(3):237–48. 10.1055/s-2007-984868 20011205 PMC2789505

[8] Buchs NC: Robotic technology: Optimizing the outcomes in rectal cancer? *World J Clin Oncol.* 2015;6(3):22–4. 10.5306/wjco.v6.i3.22 26078918 PMC4462681

[9] Intuitive Surgical, Inc - da Vinci Surgical System. 2016 Reference Source

[10] Food And Drug Administration: FDA Approves New Robotic Surgery Device. *ScienceDaily.* 2000 Reference Source

[11] Wexner SD Bergamaschi R Lacy A: The current status of robotic pelvic surgery: results of a multinational interdisciplinary consensus conference. *Surg Endosc.* 2009;23(2):438–43. 10.1007/s00464-008-0202-8 19037694

[12] Kang CY Halabi WJ Luo R: Laparoscopic colorectal surgery: a better look into the latest trends. *Arch Surg.* 2012;147(8):724–31. 10.1001/archsurg.2012.358 22508667

[13] Tsuda S Oleynikov D Gould J: SAGES TAVAC safety and effectiveness analysis: da Vinci ^(R)^ Surgical System (Intuitive Surgical, Sunnyvale, CA). *Surg Endosc.* 2015;29(10):2873–84. 10.1007/s00464-015-4428-y 26205559

[14] Kang J Hur H Min BS: Robotic coloanal anastomosis with or without intersphincteric resection for low rectal cancer: starting with the perianal approach followed by robotic procedure. *Ann Surg Oncol.* 2012;19(1):154–5. 10.1245/s10434-011-1952-4 21822556

[15] Patel VR Chammas MF Jr Shah S: Robotic assisted laparoscopic radical prostatectomy: a review of the current state of affairs. *Int J Clin Pract.* 2007;61(2):309–14. 10.1111/j.1742-1241.2006.01235.x 17263718

[16] Park EJ Kim CW Cho MS: Is the learning curve of robotic low anterior resection shorter than laparoscopic low anterior resection for rectal cancer?: a comparative analysis of clinicopathologic outcomes between robotic and laparoscopic surgeries. *Medicine (Baltimore).* 2014;93(25):e109. 10.1097/MD.0000000000000109 25437022 PMC4616378

[17] Kim HJ Choi G Park JS: Multidimensional analysis of the learning curve for robotic total mesorectal excision for rectal cancer: lessons from a single surgeon's experience. *Dis Colon Rectum.* 2014;57(9):1066–74. 10.1097/DCR.0000000000000174 25101602

[18] Smith AL Scott EM Krivak TC: Dual-console robotic surgery: a new teaching paradigm. *J Robot Surg.* 2013;7(2):113–8. 10.1007/s11701-012-0348-1 23704858 PMC3657076

[19] Shaligram A Meyer A Simorov A: Survey of minimally invasive general surgery fellows training in robotic surgery. *J Robot Surg.* 2013;7(2):131–6. 10.1007/s11701-012-0355-2 27000903

[20] Bell S Carne P Chin M: Establishing a robotic colorectal surgery programme. *ANZ J Surg.* 2015;85(4):214–6. 10.1111/ans.12817 25142978

[21] Melich G Hong YK Kim J: Simultaneous development of laparoscopy and robotics provides acceptable perioperative outcomes and shows robotics to have a faster learning curve and to be overall faster in rectal cancer surgery: analysis of novice MIS surgeon learning curves. *Surg Endosc.* 2015;29(3):558–68. 10.1007/s00464-014-3698-0 25030474

[22] Liao G Zhao Z Lin S: Robotic-assisted versus laparoscopic colorectal surgery: a meta-analysis of four randomized controlled trials. *World J Surg Oncol.* 2014;12:122. 10.1186/1477-7819-12-122 24767102 PMC4002581

[23] Xiong B Ma L Huang W: Robotic versus laparoscopic total mesorectal excision for rectal cancer: a meta-analysis of eight studies. *J Gastrointest Surg.* 2015;19(3):516–26. 10.1007/s11605-014-2697-8 25394387

[24] Pigazzi, A: Robotic Versus Laparoscopic Resection for Rectal Cancer (ROLARR). ClinicalTrials.gov: NCT01736072. Presentation at the 2015 American Society of Colorectal Surgeons Annual Meeting. Boston, MA.2015 Reference Source

[25] Scarpinata R Aly EH: Does robotic rectal cancer surgery offer improved early postoperative outcomes? *Dis Colon Rectum.* 2013;56(2):253–62. 10.1097/DCR.0b013e3182694595 23303155

[26] Keller DS Madhoun N Flores-Gonzalez JR: Effect of BMI on Short-Term Outcomes with Robotic-Assisted Laparoscopic Surgery: a Case-Matched Study. *J Gastrointest Surg.* 2016;20(3):488–93. 10.1007/s11605-015-3016-8 26704536

[27] Mirnezami AH Mirnezami R Venkatasubramaniam AK: Robotic colorectal surgery: hype or new hope? A systematic review of robotics in colorectal surgery. *Colorectal Dis.* 2010;12(11):1084–93. 10.1111/j.1463-1318.2009.01999.x 19594601

[28] Pai A Melich G Marecik SJ: Current status of robotic surgery for rectal cancer: A bird's eye view. *J Minim Access Surg.* 2015;11(1):29–34. 10.4103/0972-9941.147682 25598596 PMC4290115

[29] Kim CW Baik SH Roh YH: Cost-effectiveness of robotic surgery for rectal cancer focusing on short-term outcomes: a propensity score-matching analysis. *Medicine (Baltimore).* 2015;94(22):e823. 10.1097/MD.0000000000000823 26039115 PMC4616367

[30] Kim NK Kang J: Optimal Total Mesorectal Excision for Rectal Cancer: the Role of Robotic Surgery from an Expert's View. *J Korean Soc Coloproctol.* 2010;26(6):377–87. 10.3393/jksc.2010.26.6.377 21221237 PMC3017972

[31] Higgins RM Frelich MJ Bosler ME: Cost analysis of robotic versus laparoscopic general surgery procedures. *Surg Endosc.* 2016;1–8. 10.1007/s00464-016-4954-2 27139704

[32] Baik SH Kwon HY Kim JS: Robotic versus laparoscopic low anterior resection of rectal cancer: short-term outcome of a prospective comparative study. *Ann Surg Oncol.* 2009;16(6):1480–7. 10.1245/s10434-009-0435-3 19290486

[33] Heemskerk J de Hoog DE van Gemert WG: Robot-assisted vs. conventional laparoscopic rectopexy for rectal prolapse: a comparative study on costs and time. *Dis Colon Rectum.* 2007;50(11):1825–30. 10.1007/s10350-007-9017-2 17690936 PMC2071956

[34] Ezekian B Sun Z Adam MA: Robotic-Assisted Versus Laparoscopic Colectomy Results in Increased Operative Time Without Improved Perioperative Outcomes. *J Gastrointest Surg.* 2016;20(8):1503–10. 10.1007/s11605-016-3124-0 26966028

[35] Liao G Li YB Zhao Z: Robotic-assisted surgery versus open surgery in the treatment of rectal cancer: the current evidence. *Sci Rep.* 2016;6: 26981. 10.1038/srep26981 27228906 PMC4882598

[36] Park JS Choi GS Park SY: Randomized clinical trial of robot-assisted *versus* standard laparoscopic right colectomy. *Br J Surg.* 2012;99(9):1219–26. 10.1002/bjs.8841 22864881

[37] Park EJ Cho MS Baek SJ: Long-term oncologic outcomes of robotic low anterior resection for rectal cancer: a comparative study with laparoscopic surgery. *Ann Surg.* 2015;261(1):129–37. 10.1097/SLA.0000000000000613 24662411

[38] Biffi R Luca F Bianchi PP: Dealing with robot-assisted surgery for rectal cancer: Current status and perspectives. *World J Gastroenterol.* 2016;22(2):546–56. 10.3748/wjg.v22.i2.546 26811606 PMC4716058

[39] Collinson FJ Jayne DG Pigazzi A: An international, multicentre, prospective, randomised, controlled, unblinded, parallel-group trial of robotic-assisted versus standard laparoscopic surgery for the curative treatment of rectal cancer. *Int J Colorectal Dis.* 2012;27(2):233–41. 10.1007/s00384-011-1313-6 21912876

[40] Nayeemuddin M Daley SC Ellsworth P: Modifiable factors to decrease the cost of robotic-assisted procedures. *AORN J.* 2013;98(4):343–52. 10.1016/j.aorn.2013.08.012 24075331

[41] Trehan A Dunn TJ: The robotic surgery monopoly is a poor deal. *BMJ.* 2013;347:f7470. 10.1136/bmj.f7470 24355387

[42] Bouquet de Joliniere J Librino A Dubuisson JB: Robotic Surgery in Gynecology. *Front Surg.* 2016;3:26. 10.3389/fsurg.2016.00026 27200358 PMC4852174

[43] Liberman D Trinh QD Jeldres C: Is robotic surgery cost-effective: yes. *Curr Opin Urol.* 2012;22(1):61–5. 10.1097/MOU.0b013e32834d543f 22037320

[44] Baek SJ Al-Asari S Jeong DH: Robotic versus laparoscopic coloanal anastomosis with or without intersphincteric resection for rectal cancer. *Surg Endosc.* 2013;27(11):4157–63. 10.1007/s00464-013-3014-4 23708725

[45] Gidaro S Buscarini M Ruiz E: Telelap Alf-X: a novel telesurgical system for the 21st century. *Surg Technol Int.* 2012;22:20–5. 23225591

[46] Fanfani F Monterossi G Fagotti A: The new robotic TELELAP ALF-X in gynecological surgery: single-center experience. *Surg Endosc.* 2016;30(1):215–21. 10.1007/s00464-015-4187-9 25840895

[47] Gidaro S Altobelli E Falavolti C: Vesicourethral anastomosis using a novel telesurgical system with haptic sensation, the Telelap Alf-X: a pilot study. *Surg Technol Int.* 2014;24:35–40. 10.1016/j.juro.2013.02.2766 24706079

[48] Fanfani F Restaino S Rossitto C: Total Laparoscopic (S-LPS) versus TELELAP ALF-X Robotic-Assisted Hysterectomy: A Case-Control Study. *J Minim Invasive Gynecol.* 2016;23(6):933–8. 10.1016/j.jmig.2016.05.008 27247263

[49] Hamed A Tang SC Ren H: Advances in Haptics, Tactile Sensing, and Manipulation for Robot-Assisted Minimally Invasive Surgery, Noninvasive Surgery, and Diagnosis. *Journal of Robotics.* 2012;2012:1–14. 10.1155/2012/412816

[50] Escobar PF Haber G Kaouk J: Single-port surgery: laboratory experience with the daVinci single-site platform. *JSLS.* 2011;15(2):136–41. 10.4293/108680811X13022985132128 21902962 PMC3148858

[51] Titan Medical: Titan Medical to Unveil SPORT™ Surgical System at SAGES 2016 Annual Meeting in Boston [Press release].2016 Reference Source

[52] Paek J Lee JD Kong TW: Robotic single-site versus laparoendoscopic single-site hysterectomy: a propensity score matching study. *Surg Endosc.* 2016;30(3):1043–50. 10.1007/s00464-015-4292-9 26092018 PMC4757622

[53] Yoo HN Kim TJ Lee YY: Single-site robotic surgery in gynecologic cancer: a pilot study. *J Gynecol Oncol.* 2015;26(1):62–7. 10.3802/jgo.2015.26.1.62 25609162 PMC4302287

[54] Hompes R Rauh SM Ris F: Robotic transanal minimally invasive surgery for local excision of rectal neoplasms. *Br J Surg.* 2014;101(5):578–81. 10.1002/bjs.9454 24633833

[55] Tiwari MM Reynoso JF Lehman AC: *In vivo* miniature robots for natural orifice surgery: State of the art and future perspectives. *World J Gastrointest Surg.* 2010;2(6):217–23. 10.4240/wjgs.v2.i6.217 21160878 PMC2999241

[56] Zygomalas A Kehagias I Giokas K: Miniature surgical robots in the era of NOTES and LESS: dream or reality? *Surg Innov.* 2015;22(1):97–107. 10.1177/1553350614532549 24828382

[57] Kwak JM Kim SH: Robotic Surgery for Rectal Cancer: An Update in 2015. *Cancer Res Treat.* 2016;48(2):427–35. 10.4143/crt.2015.478 26875201 PMC4843749

[58] Bianchi PP Ceriani C Locatelli A: Robotic versus laparoscopic total mesorectal excision for rectal cancer: a comparative analysis of oncological safety and short-term outcomes. *Surg Endosc.* 2010;24(11):2888–94. 10.1007/s00464-010-1134-7 20526623

[59] D'Annibale A Morpurgo E Fiscon V: Robotic and laparoscopic surgery for treatment of colorectal diseases. *Dis Colon Rectum.* 2004;47(12):2162–8. 10.1007/s10350-004-0711-z 15657669

[60] Ghezzi TL Luca F Valvo M: Robotic versus open total mesorectal excision for rectal cancer: comparative study of short and long-term outcomes. *Eur J Surg Oncol.* 2014;40(9):1072–9. 10.1016/j.ejso.2014.02.235 24646748

[61] Yamaguchi T Kinugasa Y Shiomi A: Robotic-assisted vs. conventional laparoscopic surgery for rectal cancer: short-term outcomes at a single center. *Surg Today.* 2016;46(8):957–62. 10.1007/s00595-015-1266-4 26482845

[62] Kim YS Kim MJ Park SC: Robotic Versus Laparoscopic Surgery for Rectal Cancer after Preoperative Chemoradiotherapy: Case-Matched Study of Short-Term Outcomes. *Cancer Res Treat.* 2016;48(1):225–31. 10.4143/crt.2014.365 25779367 PMC4720082

[63] Cho MS Baek SJ Hur H: Short and long-term outcomes of robotic versus laparoscopic total mesorectal excision for rectal cancer: a case-matched retrospective study. *Medicine (Baltimore).* 2015;94(11):e522. 10.1097/MD.0000000000000522 25789947 PMC4602485

[64] Allemann P Duvoisin C Di Mare L: Robotic-Assisted Surgery Improves the Quality of Total Mesorectal Excision for Rectal Cancer Compared to Laparoscopy: Results of a Case-Controlled Analysis. *World J Surg.* 2016;40(4):1010–6. 10.1007/s00268-015-3303-2 26552907

